# List of reviewers for *Renal Failure* 2020

**DOI:** 10.1080/0886022X.2020.1858559

**Published:** 2021-03-08

The Editors of *Renal Failure* would like to thank all of our reviewers for their contribution and support during 2020. High-quality peer review is essential to the success of the Journal and we greatly appreciate the dedication of all those who have contributed to this.

A. Kaysen, George

Abdul Salim, Sohail

Abraham, Georgi

Abud, Burçin

Achanti, Anan

Adedapo, Adeolu

Aeddula, Narothama Reddy

Afkari, Rouhi

Agar, Baris U.

Agostinho Cabrita, Ana de Lurdes

Ahn, Sung Soo

Ahn, Curie

Akalin, Aysen

Akkoyun, Dursun

Akşit, Dilek

Akturk, Ibrahim Faruk

Akyuz, Aydin

Al Jurdi, Ayman

Al Musaimi, Othman

Al Shukaili, Ahmed

Ali, Ahlam

Ali, Sameh

Allam, Sridhar

Amasyali, Akin Soner

Amirteimoury, Morteza

An, Won Suk

Andrade-Oliveira, Vinicius

Antonic, Miha

Apostolou, Theophanis

Arany, Istvan

Arellano-Mendoza, Mónica Griselda

Arfian, Nur

Ari, Elif

Arslan, Mustafa

Arslan, Uğur

Arun, Oguzhan

Arya, Dharamvir Singh

Asano, Manabu

Asicioglu, Ebru

Askar, Tunay K.

Assumpcao, Lia

Ataoglu, Esra

Avci, Esin

Avramovski, Petar

Awobusuyi, Jacob

Ay, Arzu

Ayar, Yavuz

Azer, Samy A.

Aziz, Manal

Aziz, Fahad

Azoubel, Luana

Bagchi, Soumita

Bakkaloglu, Sevcan

Balamuthusamy, Saravanan

Bao, Hao

Barrera-Chimal, Jonatan

Barretti, Pasqual

Barutcu Atas, Dilek

Basher, A.

Basic-Jukic, Nikolina

Bayliss, George

Becs, Gergely

Beilstein, Christian

Beken, Serdar

Besant, Gini

Besarab, Anatole

Bevc, Sebastjan

Bingol Ozakpinar, Ozlem

Binnetoglu, Emine

Bjorneklett, Rune

Bob, Flaviu

Borulu, Ferhat

Bostan Gayret, Özlem

Bottillo, Irene

Bove, Tiziana

Bozkurt, Yasar

Brand, Serge

Branislava, Ilinčić

Brodsky, Sergey V.

Bruno, Stefania

Budisavljevic, Milos

Bunout, Daniel

Buturovic, Jadranka

Büyükcam, Fatih

Büyükkaragöz, Bahar

Cai, Guangyan

Camargo, Carlos

Canacankatan, Necmiye

Canaud, Bernard

Cander, Soner

Canetta, Pietro A. A.

Canpolat, Ugur

Canpolat, Nur

Cantekin, Işın

Cao, Dongwei

Cao, XueSen

Capusa, Cristina

Carlos, Iribarren

Castaneda, Jorge

Castellano, Giuseppe

Catar, Rusan

Chade, Alejandro

Chang, Jer-Ming

Chang, Wenxiu

Chang, Wei-wei

Charitaki, Evangelia

Charoenngam, Nipith

Chávez-Iñiguez, Jonathan S.

Chen, Aiqun

Chen, Cheng-Hsu

Chen, Chien-Liang

Chen, Guochun

Chen, Hongwei

Chen, Hui

Chen, Jin-Bor

Chen, Jun-Feng

Chen, Min

Chen, Tung-Sheng

Chen, Xiongwen

Chen, Yue

Chen, Zijin

Chen, Xiang-mei

Cheungpasitporn, Wisit

Chew, Sophia

Chi, Xinjin

Chiang, Heng-Pin

Chien, Ching-Wen

Cho, Heeyeon

Cho, Kyu Hyang

Choi, Hye Min

Choi, Eunhee

Chou, Chu-Lin

Chow, Kai Ming

Chudek, Jerzy

Chung, Sungjin

Cil, Onur

Cirrik, Selma

Cormican, Sarah

Coronado, Jorge

Costa, José Abrão

Cseprekal, Orsolya

Cui, Fangqiang

Cui, Tianlei

Cui, Wenpeng

Cui, Liyan

Cumhur cure, Medine

Cure, Erkan

Dai, Chongshan

Dai, Houyong

Dantas, Marcio

Daoud, Ahmed

Darouich, Sihem

De Francesco Daher, Elizabeth

de Menezes Montenegro, Fabio Luiz

de Moraes, Thyago Proenca

de Pont, Anne-Cornelie

Dede, Semiha

Delanaye, Pierre

Demirci, Cenk

Demirtas, Abdullah

Deng, Jin

Deng, Datong

Deveci, Köksal

Dhanapriya, Jeyachandran

Diao, Changhui

Dias, Cristiane Bitencourt

Dincel, Gungor

Ding, Jianxun

Ding, Wei

Diskin, Charles

Dixit, Mehul

Djukanovic, Ljubica

Dogan, Ibrahim

Dogra, PavitraManu

Dokur, Mehmet

Dominijanni, Sara

Donmez, Osman

Du, Xin

Duan, Shao-Bin

Dudziak, Maria

Durmaz, Mehmet Sedat

Dursun, Murat

Dusso, Adriana S

Dwivedi, Amit

Dzekova-Vidimliski, Pavlina

Eidi, Maryam

Eirini, Grapsa

Ekart, Robert

El Kassas, Mohamed

El Nakib, Gehad

Elbarbary, Hany

ElKashab, Sahier

El-Reshaid, Kamel

Elsheemy, Mohammed

Eraslan, Ersen

Eren, Huseyin

Eriguchi, Masahiro

Eroglu, Murat

Escalante, Bruno

Eskazan, Ahmet Emre

Esquivias, Elvira

Etezadi, Farhad

Fabbian, Fabio

Fan, Jie

Fan, Junming

Fang, Wei

Fang, Yu-Wei

Fanni, Daniela

Fayed, Ahmed

Feng, Zhe

Feriozzi, Sandro

Ferraioli, Giovanna

Ferreira, Viviane

Figueiredo, Ana

Figurek, Andreja

Fila, Branko

Fiorina, Paolo

Fu, Jianhui

Fu, Ping

Fu, Xuebin

Fulop, Tibor

Gadola, Liliana

Gai, Massimo

Gaipov, Abduzhappar

Gala, Agnieszka

Gal-Oz, Amir

Gan, Gin-Gin

Gao, Yan

Garfinkle, Michael

Gauthier, Alexandre

Ge, Ning

Geurts, Aron M.

Gharaibeh, Kamel

Gil, Amnon

Girisgen, Ilknur

Glassock, Richard

Gois, Pedro

Gok, Mustafa

Gökçay Bek, Sibel

Gomez, Gonzalo

Gong, Yong

Goplani, Kamal R

Gou, Shen-Ju

Goudas, Pavlos

Gu, Mingjia

Gubensek, Jakob

Gulel, Okan

Gumrukcuoglu, Hasan

Gunaydin, Zeki

Gunes, Sibel

Guo, Chun

Gupta, Ankur

Gupta, Dr. Krishan Lal

Gyamlani, Geeta

Halwachs-Baumann, Gabriele

Hamrahian, Mehrdad

Han, Fei

Han, Mei

Hanset, Nicolas

Haroon, Sabrina Wong Peixin

Hayiroglu, Mert Ilker

He, Fan

He, Lijie

He, Peng

He, Xiaoshun

He, Yunqiang

He, Qiqi

He, Linhua

Heidari, Ghobad

Hernández Garduño, Eduardo

Heybeli, Cihan

Hidaka, Sumi

Hiki, Masaru

Hogan, Susan

Holick, Michael F

Hong, Quan

Horinouchi, Yuya

Horvatic, Ivica

Hoscan, Mustafa Burak

Hosojima, Michihiro

Hosseinzadeh-Attar, Mohammad Javad

Hsu, Heng Jung

Hu, Bo

Hu, Henglong

Hu, Jie

Hu, Zhangxue

Huang, Gang

Huang, Kun

Huang, Wei-Lieh

Huang, Chien-Wei

Hung, Ming-Jui

Iguchi, Naoya

Ikeda, Yasumasa

Ilgen, Ufuk

Inaguma, Daijo

Inal, Volkan

Indridason, Olafur S.

Io, Hiroaki

Ishii, Hideki

Ishiyama, Katsuya

Ishizu, Akihiro

Isitmangil, Gulbu

Ismail, Ammar

Ismail, Gener

Iwasaki, Yoshiko

Jalandhara, Nishant

Jeloka, Tarun

Jha, Vivekanand

Jiang, Shanqun

Jiang, X. -Y.

Jiang, Yuteng

Jiang, Yanfang

Jong, Gwo-Ping

Jorgetti, Vanda

Joshi, Kusum

Julian, Bruce A.

Junglee, Naushad

Justa, Shivali

Justo, Dan

Kadihasanoglu, Mustafa

Kaewput, Wisit

Kahraman, Dogan

Kajbafzadeh, Abdol Mohammad

Kaminska, Dorota

Kang, Kyung Pyo

Kang, Seok Hui

Kaplan, Halil mahir

Karaboyas, Angelo

Karabulut, Ahmet

Karan, Mehmet Akif

Kasapoglu, Benan

Kaskel, Frederick

Kataria, Ashish

Katavetin, Pisut

Kawano, Mitsuhiro

Kaya, Yasemin

Kaygin, Mehmet Ali

Kaypakli, Onur

Kazancioglu, Rumeyza

Kebapci, Nur

Kei, Anastazia

Khalaf, Mohamed

Kiessling, Arndt-Holger

Kilis-Pstrusinska, Katarzyna

Kim, Gheun-Ho

Kim, Ki-Young

Kim, Won

Kinashi, Hiroshi

Kirsch, Alexander H.

Kıvrak, Elfide Gizem

Klopotowski, Mariusz

Koch, Christian

Kocyigit, Ismail

Kohli, Harbir

Komaba, Hirotaka

Kopitko, Csaba

Korkmaz, Hasan

Koser, Murat

Koukoulaki, Maria

Kreepala, Chatchai

Krieter, Detlef H.

Kuczera, Piotr

Kuma, Akihiro

Kumar, Jitendra

Kuo, Ko-Lin

Kuriyama, Satoru

Kurtul, Alparslan

Kwon, Osun

Kwon, Soon Kil

Kwon, Young Joo

Lai, Silvia

Lai, Carlo

Larsson, Anders

Laucyte-Cibulskiene, Agne

Lee, Cheng-Chia

Lee, Kuo-Hua

Leeaphorn, Napat

Lengvárszky, Zsolt

Lerant, Anna

Letachowicz, Krzysztof

Leung, Nelson

Li, Can

Li, Huihui

Li, Jianying

Li, S.

Li, Weizu

Li, Xiaoran

Li, Xinli

Li, Xudong

Li, Yi

Li, Ying

Li, Zhanzhan

LI, Guisen

Li, Mingcheng

Li, Wenxiong

LI, Yuehong

Liang, Kelly V.

Liang, Xinling

Liern, Miguel

Lim, Soo Kun

Lin, Hongli

Lin, Tao

Lionaki, Sophia

Liou, Hung-Hsiang

Liu, Longshan

Liu, Na

Liu, Tongqiang

Liu, Wenhu

Liu, Yuyuan

Liu, Zhongyan

Locatelli, Francesco

Long, Haibo

Lu, Chien-Lin

Lu, Kuo-Cheng

Lu, Xiaohan

Lukrafka, Janice

Lun, Hengzhong

Luo, Ping

Schetz, Marie

Ma, Yuan

Ma, Xiaofen

Mahajan, Sandeep

Malhotra, Sheetal

Mallamaci, Francesca

Mallios, Alexandros

Malyszko, Jolanta

Markić, Dean

Marques, Igor

Marques, Maria

Marti, Hans-Peter

Maruniak-Chudek, Iwona

Maruyama, Yukio

Masakane, Ikuto

Matsumoto, Yoshihiro

Mazhar, Kehkashan

Meier, Clemens M.

Mendonca, Satish

Meng, Juan

Mercadal, Lucile

Merlino, Giovanni

Miao, Liying

Michael, Ashour

Milosevic, Danko

Misra, Sanjay

Miura, Kenichiro

Mizerska-Wasiak, Magorzata

Mizuno, Masashi

Mobarra, Naser

Molnar, Miklos Zsolt

Molsted, Stig

Moosavi, Seyed

Morishita, Yoshiyuki

Morrone, Luigi

Mou, Shan

Mushahar, Lily

Myserlis, Paul

Na, Nin

Nair, Sreeja

Nakagawa, Naoki

Naqvi, Rubina

Nasif, Wesam

Nayman, Alaaddin

Ndlovu, Kwazi C. Z.

Neciolu Orken, Dilek

Neugarten, Joel

Nghia, Vu

Nie, YuXin

Nigam, Lovelesh

Nigwekar, Sagar

Nikvarz, Naemeh

Nisari, Mehtap

Nishino, Tomoya

Nochaiwong, Surapon

Nowicki, Michał

Nurmohamed, Shaikh

Obrisca, Bogdan

Odiatis, Christoforos

Ogata, Hiroaki

Oh, Dong-Jin

Okamoto, Takayuki

Okamoto, Teppei

Oliveira, E. A.

Omboni, Stefano

Oneib, Bouchra

Orisakwe, Orish Ebere

Ossareh, Shahrzad

Ouyang, Chun

Oygar, Duriye

Ozaki, Iwata

Ozen, Nurten

Ozkan, Gulsum

Pajek , Jernej

Pan, Min

Panagiotou, Angeliki

Pandak, Nenad

Pandya, Vaidehi

Pape, Lars

Parlakpinar, Hakan

Parv, Florina

Pavlovic, Drasko

Paydas, Saime

Pedraza-Chaverri, José

Pedroso, Jose

Pehlivan, Sacide

Pei, Juan

Pembegul Yigit, Irem

Peng, Ai

Peng, Fenfen

Peng, Yu-Sen

Peng, Linlin

Perazella, Mark

Perez, Renata

Perez Morales, Rosa Elena

Perna, Annalisa

Petejova, Nadezda

Pethő, Ákos

Petras, Dimitrios

Petrauskiene, Vaida

Petrova, Iliyana

Pike, Mindy

Pirozzi, Nicola

Pitt, Bertram

Podestà, Manuel

Ponce, Daniela

Pottel, Hans

Praga, Manuel

Qi, Min-You

Qiao, Xi

Qin, Shu-Lan

Qiu, Ming-zhu

Quadri, Syed

Radanova, Maria

Radovic, Milan

Ralib, Azrina

Ramachandran, Raja

Ramratnam, Mohun

Randjelovic, Pavle

Rangan, Gopala

Raptis, Vassilios

Rathore, Hassaan A

Raucci, Angela

Ravn, Bo

Ren, Hongqi

Rewa, O. G.

Rocha, Paulo

Rodrigues, Juliana

Rodrigues, Vinícius

Roozbeh, Jamshid

Rossi Feliciano, Marcus Antonio

Rotondi, Silverio

Rroji, Merita

Saad, M-A A. A.

Sabry, Alaa

Sadeghnia, Hamid

Saedi, Babak

Saito, Takao

Sakai, Ken

Sakkas, Giorgos

Sancak, Eyup

Sanchez-Lozada, L. Gabriela

Sayin, Burak

Schneditz, Daniel

Schönmarck, Ulf

See, Lai-Chu

Seeman, Tomas

Segelmark, Mårten

Seifi, Behjat

Seixas, Emerson

Selcuk, Nedim

Serafini, Gianluca

Sethi, Gautam

Seyler, Lucie

Sezer, Mehmet

Shacham, Yacov

Shang, Shun Lai

Shanmugam, Vijay

Shapiro, Joseph

Sharaf El Din, Usama

Sharma, Madhulika

Sharma, Sadhana

Sheikhbardsiri, Hojjat

Shen, Bo

Shengyou, Yu

Shiao, Chih-Chung

Shigematsu, Takashi

Shinkov, Alexander

Sikole, Aleksandar

Singer, Eric A.

Śledziński, Tomasz

Heyman, Samuel N.

Soliman, Karim

Song, Yu-Huan

Sousa, Clemente Neves

Soysal, Pinar

Srivali, Narat

Stauder, Adrienne

Stavroulopoulos, Aristeidis

Stefan, Gabriel

Stepien, Ewa

Sterner, Gunnar

Steubl, Dominik

Stompór, Tomasz

Stompór, Tomasz

Storr, Markus

Strohmaier, Walter Ludwig

Suchal, Kapil

Sugi, Motohiko

Sugiyama, Noriyuki

Suleyman, Zeynep

Sumer, Ismail

Sun, In O.

Sun, Jing

Sung, Junne-Ming

Susantitaphong, Paweena

Suzuki, Yusuke

Suzuki, Hitoshi

Szabo, Laszlo

Szabo, Eva

Taguchi, Takafumi

Taheri, Saeed

Tamura, Kouichi

Tan, Ying

Tanaka, Hiroshi

Tang, Zheng

Tang, Rong

Tanimoto, Minako

Tao, Xingjuan

Tapolyai, Mihaly

Tesar, Vladimir

Thajudeen, Bijin

Thamcharoen, Natanong

Thibaudin, Damien

Thongprayoon, Charat

Tian, Jun-Ping

Tisljar, Miroslav

Tombak, Anil

Tong, Fei

Trakarnvanich, Thananda

Tranche, Salvador

Trovato, Guglielmo

Tsai, Yi-Chun

Tsai, Shangfeng

Tseke, Paraskevi

Tsourous, George

Tsujita, Makoto

Tu, Xiao

Turgut, Faruk

Uçar, Mehmet Ali

Ueno, Yuji

Ullah, Kifayat

Ullian, Michael

Urushihara, Maki

Uygun, Saime

Van Buren, Peter N.

Vanotti, Sandra

Varma, Ravi Prasad

Vega, Almudena

Velioglu, Arzu

Venneri, Mary Anna

Verbrugge, Frederik H.

Verma, Himanshu

Vujkovac, Bojan

Vychytil, Andreas

Wakino, Shu

Waly, Mostafa

Wan, Xin

Wang, Changxi

Wang, Guyan

Wang, Hao

Wang, Jiawu

Wang, Jinwei

Wang, Ming-jie

Wang, Qiuyue

Wang, Xinchen

Wang, Yanhong

Wang, Ningning

Wang, Weiming

Ward, Richard A.

Weinhandl, Eric

Wen, Mei

Wesseling, Catharina

Widgren, Jennie Lönnbro

Wiecek, Andrzej

Wilson, Samuel Eric

Wu, Gang

Wu, Tai-Te

Xiao, Cheng-cheng

Xiong, Mingxia

Xu, Dongmei

Xu, Guangtao

Xu, Guoshuang

Xu, Jiarui

Xu, Tao

Xu, Yan

Yadav, Raj

Yamada, Shunsuke

Yamamoto, Hiroyasu

Yan, Jianyun

Yan, Ming-Tso

Yang, Chun

Yang, Guang

Yang, Li

Yang, Qiongqiong

Yang, Xiao

Yang, Yanlang

Yang, Yingying

Yang, Fan

Yang, Won Seok

Yi, Tiegang

Yıldız, Fatih

Yilmaz, Akar

Yılmaz, Mümtaz

Yokoyama, H

Yong, Tuck

Yu, Xiaofang

Yu, Xiaoyang

Yu, Zihua

Yuan, Xiao-peng

Yuksel, Selcuk

Zacharoulis, Dimitrios

Zamani, Nasim

Zanon, Jeferson

Zayed, Bahaa

Zeng, Ming

Zhan, Xiaojiang

Zhang, Dongliang

Zhang, Guangyuan

Zhang, Jianbin

Zhang, Jiandong

Zhang, Wei

Zhang, Zaiqing

Zhang, Zhongheng

Zhang, Xiaojuan

Zhang, Haitao

Zhang, Hao

Zhao, Fusheng

Zhao, Huiping

Zhao, Li-Jun

Zhe, Li

Zheng, Cai-Mei

Zheng, Chenfei

Zhou, Chao

Zhou, Fu-de

Zhou, Yilun

Zhou, Li

Ziamajidi, Nasrin

Zilberman-Itskovich, Shani

Zoccali, Carmine

Zou, Jianzhou

Zou, Xiangyu

Zsom, Lajos

